# Effectiveness of Prior Use of Beta-Blockers for Preventing Adverse Influences of Severe Hypoglycemia in Patients With Diabetes: An Observational Study: Erratum

**DOI:** 10.1097/01.md.0000480960.09826.a5

**Published:** 2016-02-12

In the article “Effectiveness of Prior Use of Beta-Blockers for Preventing Adverse Influences of Severe Hypoglycemia in Patients With Diabetes: An Observational Study”,^1^ which appeared in Volume 94, Issue 39 of *Medicine*, the title of Table 2 contained an error. The article has since been corrected online.
